# Homœopathy and Cholera

**Published:** 1849-07

**Authors:** 


*Homœopathy and Cholera.*—Since the appearance of Cholera in this city, our homœopathic friends have laudably endeavored to do their part in enlightening the community in regard to its prevention and cure. Hence we have had through the daily papers, various private and *official* documents on the subject. We have for some time been aware, that in this country at least, true homœopathy no longer existed except in name; but we were not quite prepared for so frank an acknowledgement of the fact as has been made in the documents referred to. Thus we are told in the communication from the committee appointed by the "Homœopathic Physicians' Society of New York," that the proper remedies for cholera are *Cuprum Metalicum* or *Veratrum* in the first stage, and if the patient becomes bad the *Spirits* of *Camphor* must be resorted to. Yes, the veritable "*Spirits* of *Camphor*," not the 30th dilution, ner the 61st trituration, but *spirits* of camphor, and that in doses of three drops repeated every few minutes if the symptoms are urgent. The committee making this report is composed of six or eight of the most prominent homœopathists of this city. Their names may be found in the daily New York Tribune for the 5th inst. Notwithstanding the boasted certainty and specific nature of homœopathic remedies, there seems still to be some differences of opinion in regard to the true homœopathic remedy for the cholera. Hence, in the Tribune for June 8th, we find a communication from Charles J. Hempel, who, though a member of the New York Homœopathic Physicians' Society, yet takes the liberty to differ from the report of the said committee. He regards the cuprum, the veratrum, and the camphor, only as palliatives, while the Aconitum Napellus furnishes the only true cholera specific. The following are his directions for its use, viz:

"As soon as the diarrhœa sets in, with or without cramps in the stomach and bowels, with or without vomiting, coldness of the extremities, etc., dissolve 5 drops of the *tincture of Aconite* in 10 table-spoonsful of clear Croton water, and take two tea-spoonfuls every half hour, until an improvement sets in; then continue every two hours until you feel entirely well. Eat very little, and only light food, gruels, weak tea and toast, etc.

If the diarrhœa should be very bad, attended with or without cramps in the bowels, spasms in the extremities, vomiting, or if the paroxysms should set in immediately with great force, dissolve 10 drops of the tincture of Aconite in ten table-spoonfuls of water, and give the patient 2 tea-spoonfuls every five minutes until the pulse improves, the extremities become warm, and a moisture is perceived on the skin; then continue every 20 minutes until the improvement is strikingly manifest, and finally continue every two hours until the patient is entirely recovered."

There it is, real, genuine, *Crude Tincture of Aconite*, in doses too amounting to nearly one drop every *five* minutes, or *ten* drops every hour

There is no *dilution*, no *trituration* about it; for he tells us that he uses the *tincture* prepared after Pereira's formula. And in regard to the dose, it should certainly satisfy any allopathist in the country. Pereira himself directs only *five drops three times a day.*

If we had been desirous of proposing a plan of treatment diametrically opposed to the so called principles of homœopathy in every particular, we could not have accomplished our object better than by adopting the course here recommended by the first homœopathists in this city. Is there the remotest possible *similarity* between the symptoms induced by camphor, and those of cholera? Is there even an *approximation*, between *three* drop doses of Spts. Camphor, or *one* drop doses of Tinct. Aconite, *every five minutes*, and the smelling, or even taking of a *pellet* of the 30th *dilution* of either? Alas! for the doctrines of *attenuation* and *Similia Similibus.* Well may our friend Kirby, of the American Journal of Homœopathy, exclaim that, "a mongrel in medicine, of all men is the most inconsistent.—*Annalist. Editorial.*

